# Full-endoscopic versus conventional microsurgical therapy of lumbar disc herniation: a prospective, controlled, single-center, comprehensive cohort trial (FEMT-LDH trial)

**DOI:** 10.1186/s13063-022-06892-8

**Published:** 2022-12-07

**Authors:** Babak Saravi, Sara Ülkümen, Sebastien Couillard-Despres, Frank Hassel, Gernot Lang

**Affiliations:** 1 Department of Spine Surgery, Loretto Hospital, Freiburg, Germany; 2 Department of Orthopedics and Trauma Surgery, Medical Centre - Albert-Ludwigs-University of Freiburg, Faculty of Medicine, Albert-Ludwigs-University of Freiburg, Hugstetterstrasse 55, 79106 Freiburg, Germany; 3 Institute of Experimental Neuroregeneration, Spinal Cord Injury and Tissue Regeneration Center Salzburg (SCI-TReCS), Paracelsus Medical University, 5020 Salzburg, Austria

**Keywords:** Disc prolapse, Minimal-invasive, Randomized, Trial, Endoscopic, Spinal stenosis, Disc herniation

## Abstract

**Background:**

Lumbar disc herniation is one of the leading causes of chronic low back pain. Surgery remains the therapy of choice when conservative approaches fail. Full-endoscopic approaches represent a promising alternative to the well-established microsurgical technique. However, high-grade evidence comparing these techniques is still scarce.

**Methods:**

Patients presenting with lumbar disc herniation will be included. The intervention group will obtain full-endoscopic disc decompression, whereas the control group will be treated by microsurgical disc decompression. We will apply a comprehensive cohort study design involving a randomized and a prospective non-randomized study arm. Patients who do not consent to be randomized will be assigned to the non-randomized arm. The primary outcome will be the Oswestry Disability Index (ODI). Secondary outcomes involve the visual analog scale (VAS) of pain and the SF-36 health questionnaire. Furthermore, clinical characteristics including duration of hospital stay, operation time, and complications as well as laboratory markers, such as C-reactive protein, white blood cell counts, and interleukin 6 will be determined and compared.

**Discussion:**

This study will significantly contribute to the current evidence available in the literature by evaluating the outcome of the full-endoscopic technique against the gold standard for lumbar disc herniation in a clinically relevant study setup. Additionally, the study design allows us to include patients not willing to be randomized in a prospective parallel study arm and to evaluate the impact of randomization on outcomes and include. The results could help to improve the future therapy in patients suffering from lumbar disc herniation.

**Trial registration:**

This study was prospectively registered in The German Clinical Trials Register (DRKS), a German WHO primary registry, under the registration number: DRKS00025786. Registered on July 7, 2021.

## Administrative information

Note: the numbers in curly brackets in this protocol refer to SPIRIT checklist item numbers. The order of the items has been modified to group similar items (see http://www.equator-network.org/reporting-guidelines/spirit-2013-statement-defining-standard-protocol-items-for-clinical-trials/).

**Table:** 

Title {1}	Full-Endoscopic versus conventional Microsurgical Therapy of Lumbar Disc Herniation: a prospective, controlled, single-center, comprehensive cohort trial (FEMT-LDH trial)
Trial registration {2a and 2b}.	This study was prospectively registered in The German Clinical Trials Register (DRKS), a German WHO primary registry, under the registration number: DRKS00025786
Protocol version {3}	First published version: 07/07/2021
Funding {4}	Loretto Hospital Freiburg, Germany: provided administrative support, surgeons, and research scientists employee salaries. Joimax GmbH: funding of costs associated with laboratory marker assessments, funding of ethical approval costs, funding of publication costs, funding of congress expenses related to the project
Author details {5a}	Babak Saravi^1,2^*, Sara Ülkümen^1,2^, Sebastien Couillard-Despres^3^, Frank Hassel^1^, Gernot Lang^2^ ^1^Department of Spine Surgery, Loretto Hospital, Freiburg, Germany ^2^Department of Orthopedics and Trauma Surgery, Medical Centre - Albert-Ludwigs-University of Freiburg, Faculty of Medicine, Albert-Ludwigs-University of Freiburg, Hugstetterstrasse 55, 79106 Freiburg, Germany ^3^Institute of Experimental Neuroregeneration, Spinal Cord Injury and Tissue Regeneration Center Salzburg (SCI-TReCS), Paracelsus Medical University, 5020 Salzburg, Austria *Corresponding author FH, GL and SCD are the Chief Investigators; they conceived the study, led the proposal and protocol development. GL, BS, SCD and SU contributed to the study design and development of the proposal. GL, BS and SCD were the lead trial methodologist. BS and SU wrote and edited the present protocol. All authors read and approved the final manuscript.
Name and contact information for the trial sponsor {5b}	-Joimax GmbH, Raumfabrik 61, 76227 Karlsruhe, Germany -Loretto Krankenhaus Freiburg, Mercystraße 6-14, 79100 Freiburg, Germany
Role of sponsor {5c}	Loretto Hospital Freiburg, Germany: provided administrative support, surgeons, and research scientists. employee salaries. Joimax GmbH: funding of costs associated with laboratory marker assessments, funding of ethical approval costs, funding of publication costs, funding of congress expenses related to the project. Funder and sponsors had no influence on study design. Funder and sponsors will not have an influence on collection, management, analysis, and interpretation of data, writing of the report, and the decision to publish the study protocol and/or study results.

## Introduction

### Background and rationale {6a}

Lumbar disc herniation is one of the leading causes of chronic back pain and has a lifetime prevalence of up to 3.5% in under 35 years old patients in western countries [1]. In 45- to 55-year-olds, the prevalence increases to over 20% [2]. The pathophysiology includes traumatic or disease-related loss of integration of the annulus fibrosus with subsequent herniation of soft nucleus-pulposus material. This herniation results in a prolapse into the subligamentous or epidural space with or without nerve compression. The nerve compression leads to the development of pain, which the patients report on medical examinations (intermittent or chronic lumbar pain usually precedes the acute event of a herniated disc) [3]. Conservative therapy is the therapy of choice in over 80% of patients with a lumbar disc herniation as the first event. It is based on bed rest, pain medication, muscle relaxants, and the avoidance of bad posture. All conservative measures aim to improving symptoms [4]. If the conservative treatments are exhausted, if cone or caudal symptoms are present, or in the case of rapidly progressive and severe neurological deficits, surgical therapy is indicated. There are several surgical methods used to treat herniated discs effectively.

The standard operative therapy is microsurgical sequestrectomy using an operating microscope [5]. The use of microsurgical techniques has reduced tissue damage and its consequences [6]. Although this operative technique has proven itself in clinics, continuous optimization of the operative procedures should be attempted. The aim of a new approach must be to minimize the trauma and its negative long-term consequences.

Minimally invasive techniques can reduce tissue damage and its consequences. Endoscopic operations have become standard in many areas. The most widely used full-endoscopic procedure in patients with lumbar disc disease is transforaminal surgery [7]. Laser and bipolar cauterization can be used. The removal of the intra- or extraforaminal sequestered material is technically possible. The resection of the sequestered nucleus pulposus within the spinal canal, i.e., a retrograde resection performed intradiscally through the existing annular defect, has already been described [8]. Nevertheless, difficulties in adequately resecting herniated discs within the spinal canal cannot always be ruled out. With lateral access, the spinal canal can be better reached with continuous visualization. However, the bony circumference of the foramen and exiting nerve can limit labor mobility and excision. In addition, the pelvis and abdominal structures can block access. Therefore, there may be restrictions utilizing the transforaminal access. The full-endoscopic interlaminar approach was developed to treat pathological situations that could not be successfully performed with the transforaminal technique [9].

Based on the results of a recently carried out systematic review, the full-endoscopic discectomy is regarded as one of the most effective treatment methods, which is supposed to be on a par with the gold standard of microsurgical surgical procedures [7]. However, the authors requested further prospective randomized studies to validate the results. The advantages of the full-endoscopic approach compared to open and microsurgical surgical procedures were, among other things, less bleeding, fewer complications, and faster recovery or an early return to everyday life. At present, there is not yet sufficient evidence regarding iatrogenic muscle trauma in the comparative examination of the two methods. The comparison of muscle damage parameters (surrogate markers) in the blood of the treating patients could provide further information about the outcome of the existing therapies.

Furthermore, patient-oriented outcomes, such as the perception of pain and limitations in everyday life, are relevant and need to be evaluated in affected patients. Finally, the available evidence does not sufficiently report the surgeon’s experience level and the number of surgeons involved when comparing both techniques, representing a significant limitation [10, 11]. The study aims to compare the results of lumbar discectomies using the full-endoscopic technique via the interlaminar and transforaminal access with those of the conventional microsurgical technique, particularly with regard to iatrogenic muscle trauma and patient-related outcome measures. Notably, one surgeon who is highly experienced in both methods will perform the surgery. This investigation will take place as a “comprehensive cohort study,” including a randomized and non-randomized study arm comparing the endoscopic versus microsurgical decompression to provide a high level of evidence. Further, this approach allows us to evaluate the impact of randomization on study outcomes.

### Objectives {7}

We sought to compare the full-endoscopic versus microsurgical technique for the decompression of lumbar disc herniation, focusing on patient-reported outcome measures, clinical characteristics, and laboratory marker evaluations. Patient-reported outcome measures will be evaluated preoperatively, immediately postoperatively, after 24 h, 48 h, 3 weeks, 1 year, 2 years, and 5 years postoperatively to provide the necessary and currently lacking long-term data.

### Trial design {8}

**Fig. 1:**
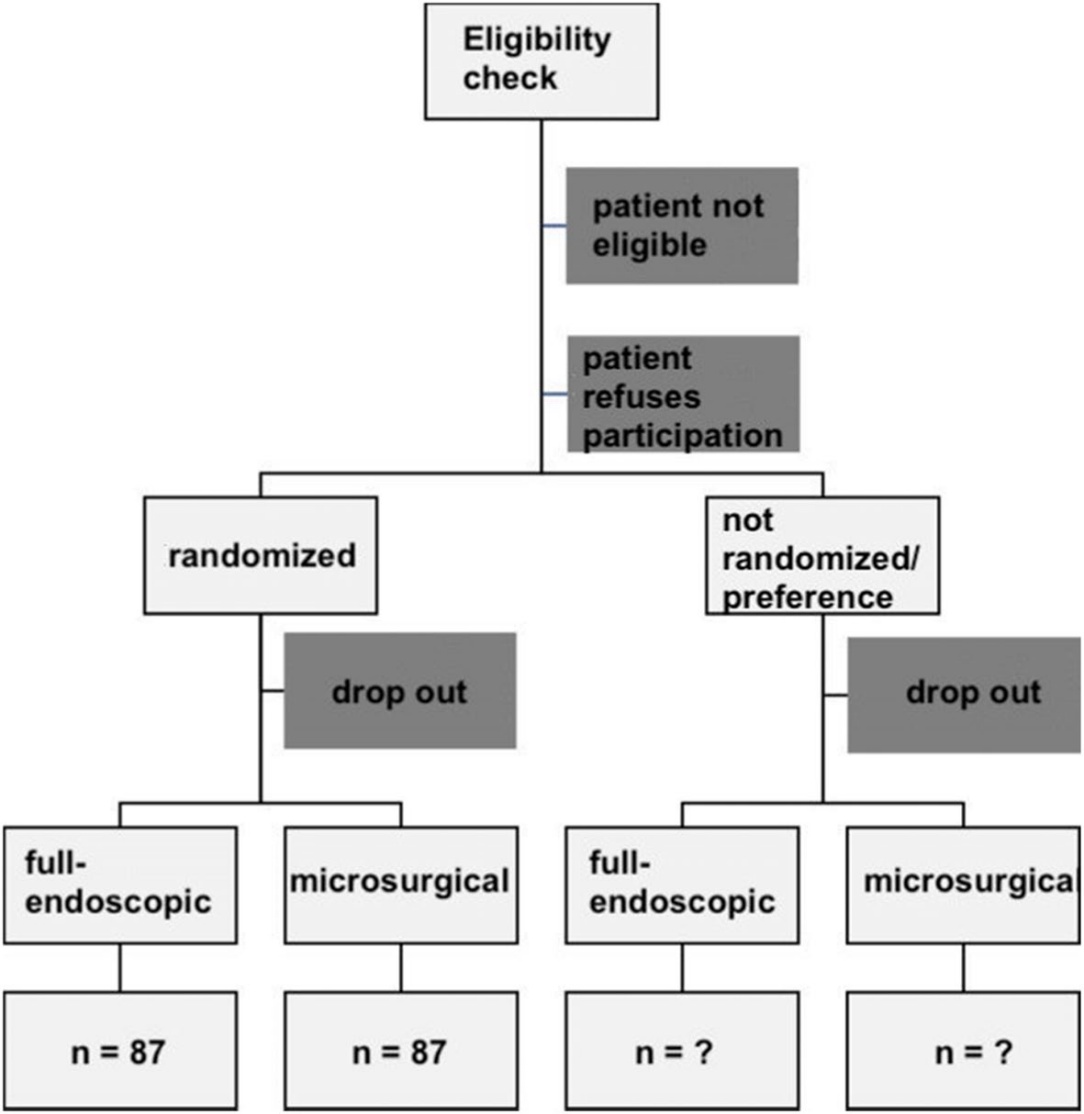
Illustration of the study design

We will perform a comprehensive cohort trial involving a randomized and a prospective non-randomized study arm, each including intervention (full-endoscopic surgery) and control (microsurgical decompression) groups (superiority framework). Patients who do not consent to be randomized will be assigned to the non-randomized arm. Figure 1 illustrates the study design.

## Methods: participants, interventions, and outcomes

### Study setting {9}

Patients will be recruited at the Loretto Hospital Freiburg, Freiburg, Germany. Surgery, evaluations, follow-ups, and data analysis will be performed at the same institution.

### Eligibility criteria {10}

Inclusion criteria:

- Gender: Both male and female
- Minimum age: 18 years
- Maximum age: 75 years
- Patients for whom the medical indication for lumbar disc surgery is given and conservative therapy was unsuccessful

Exclusion criteria:

- Patients under 18 years of age
- Patients incapable of giving consent
- Patients who contact the Loretto Hospital in advance with the request of one of the two surgical methods (these patients will be assigned to the respective non-randomized group if they consent to participate in the study)
- Patients who do not agree to follow-up appointments 3 weeks, 1 year, 2 years, and 5 years post-op
- Breastfeeding, pregnant, or childbearing women who plan to become pregnant while participating in the study
- Massively sequestered disc prolapse
- Prolapse in the upper lumbar region (L2 or above)
- Patients with previous prolapse and revision surgery
- Prolapse affects several segments
- Patients ≥ 120 kg
- Patients with an active infection process
- Patients with other serious, especially malignant, diseases
- Patients with other severe spinal disorders, such as osteoarthritis, osteopenia, osteoporosis, or osteomalacia
- Patients with congenital malformations of the spine

Eligibility criteria for the surgeon: Intervention will be performed by one spine surgeon with at least 5 years of clinical experience in both surgical techniques.

### Who will take informed consent? {26a}

Informed consent will be obtained from FH and GL. The informant consent includes an extensive explanation of the surgery, the study design, the pros and cons of the techniques, and a detailed description of how data are handled. Patients will further be informed that their participation in a non-randomized study arm is possible if they refuse to be randomized or already have a preference regarding the surgical technique. Patients will receive a copy of the patient informed consent and get sufficient time to solve their doubts.

### Additional consent provisions for collection and use of participant data and biological specimens {26b}

Not applicable as no additional biological specimens will be analyzed. Blood samples are regularly taken during surgery and postoperatively and do not require additional consent.

### Interventions

#### Explanation for the choice of comparators {6b}

We will compare the full-endoscopic disc decompression versus microsurgical disc compression for lumbar spinal stenosis. Our study aims to test the superiority of full-endoscopic decompression against the current gold standard.

#### Intervention description {11a}

**Fig. 2:**
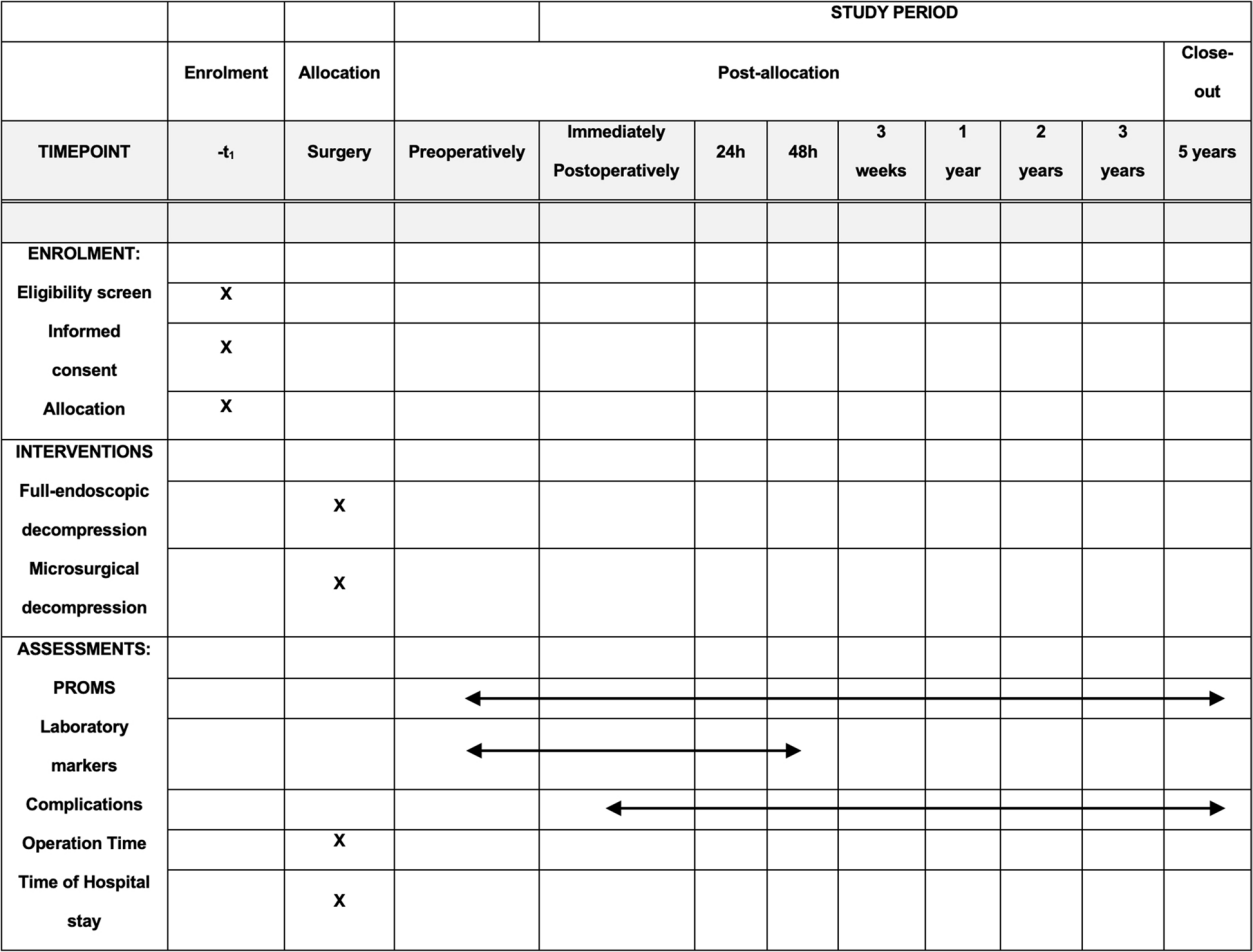
Illustration of SPIRIT study timeline. Patient-reported outcome measures (PROMs), Oswestry Disability Index (ODI), SF-36 health survey, and visual analog scale (VAS) of pain

Upon enrollment in this clinical study, medical history will be recorded, and patients will undergo a comprehensive medical examination. This includes, in particular, a physical examination, an assessment of pain and symptoms, a neurological examination, an x-ray of the lower back (lumbar spine), computed tomography, or magnetic resonance tomography (if not recorded otherwise in the 6 months before the date of the surgery). These imaging procedures are part of the conventional treatment for back operations at our hospital and are also carried out regardless of participation in the study. Patients will provide blood samples preoperatively, immediately postoperatively, and after 48 h postoperatively. These examinations are part of the conventional treatment for back operations and are also carried out in the hospital regardless of participation in the study. However, in addition to the blood markers usually obtained and assessed in the study (C-reactive protein, white blood cell count, creatine kinase), we will also include interleukin 6 as a marker for inflammation and tissue damage. Complication rates seen during the surgery and postoperative complications will be determined. Time of hospital stay will be recorded. Follow-up examinations will be performed in time intervals illustrated in Fig. 2. For patient-reported outcome measures (PROMs), patients will be asked to fill out the respective questionnaire in predefined time intervals at our hospital. The skin incision for the gold standard (microsurgical technique) is about 3 to 5 cm long and depends mainly on the length of the narrowed nerve path. The parts of the intervertebral discs that exert pressure on the nerves and/or the spinal cord are removed with special instruments advanced through a trocar (sleeve) or spreader until the herniated disc is reached. The operation is performed utilizing a surgical microscope. After removing all the instruments, the skin is sutured.

“Full endoscopic” means that the operation is carried out exclusively using an endoscope. The skin incision is about 0.8 and a maximum of 1 cm long. The parts of the intervertebral discs that exert pressure on the nerves and/or the spinal cord are removed with special instruments inserted through the working channel integrated into the endoscope until the herniated disc is reached. A camera is incorporated in the endoscope so that the operation is carried out under visual control. After removing all the instruments, the skin is sutured.

#### Criteria for discontinuing or modifying allocated interventions {11b}

Discontinuation of the intervention will be applied if the intervention failed to allow sufficient treatment of the pathology during surgery (switch to the microsurgical technique (“conversion”) and upon participants' request at any time.

#### Strategies to improve adherence to interventions {11c}

Our strategy to improve adherence to the follow-up monitoring includes a face-to-face adherence reminder session with the surgeon (FH) at the regular postoperative follow-up visits. Furthermore, we will ask patients during the visits how they can be best contacted (e.g., telephone, WhatsApp, SMS). The approach to remind patients to attend the follow-up visits for questionnaires will depend on their preferred way to be contacted.

#### Relevant concomitant care permitted or prohibited during the trial {11d}

Patients are asked to inform the doctor regarding newly diagnosed diseases potentially affecting the outcomes of interest. Decision on excluding patients suffering from relevant diseases that could affect outcome assessments will be performed by the surgeon (FH) after contacting the doctor who diagnosed the disease and discussion with at least two additional research members of the study.

#### Provisions for post-trial care {30}

There will be no provisions and no compensation to those who suffer harm from trial participation.

#### Outcomes {12}

Primary target variables:

Patient-related outcome measures (PROMs):

- Oswestry Disability Index (ODI)

Secondary target variables:

- Health questionnaire SF-36
- Pain intensity using the visual analog scale (VAS)
- Duration of the hospital stay (in days)
- Postoperative complications
- Duration of opioid use
- Operating time (in minutes)
- Re-admission within 3 weeks, 1 year, 2 years, 5 years postoperatively
- Blood parameters for evaluating muscle damage:
  - Creatine kinase (CK)
  - Glomerular filtration rate
  - Myoglobin (Mb)
  - Erythrocyte sedimentation rate
  - Creatine kinase: aspartate aminotransferase quotient
  - Procalcitonin (PCT)
  - C-reactive protein (CRP)
  - White blood cell count (WBC)
  - Interleukin (IL)-6

Renal parameters (e.g., creatinine clearance, glomerular filtration rate) will be measured to assess the influence of renal function on outcome measures, as creatinine kinase levels and myoglobin levels might be influenced by renal function. The severity of pain is determined using the visual analog scale (VAS). The scale is a 10-cm-long straight line, at the ends of which the extreme points of the pain to be measured are noted (0 cm = no pain, 10 cm = worst imaginable pain). The patient is asked to mark the intensity of his pain on the line with a pen [8].

The VAS is collected on the examination days before treatments (e.g., physiotherapy) are carried out. To assess the change in pain symptoms in the patients, the VAS values of the study groups at the time of the examination are compared and compared with statistical methods. The minimal clinically relevant reduction is given for input values above 30% [8].

The Oswestry Disability Index (ODI) is a questionnaire for evaluating functional disorders caused by back pain. The German version published in 2006 [9], the ODQ-D (Oswestry Disability Questionnaire- German), is scientifically recognized, and researchers regularly use it in back pain studies. The questionnaire is divided into 10 sections, each with 6 possible answers with point values of 0–5 points. The most appropriate answer should be ticked. As a result, the number of points achieved is set in relation to the maximum number of points and given as a percentage. Based on the results, the following judgments are made: 0–20% equals minimal, 21–40% equals moderate, 41–60% equals severe, 61–80% equals crippled, and 81–100% equals bed-bound or patients exaggerating their symptoms. The ODI will be measured as the primary outcome. The following time points will be assessed for the ODI: preoperatively, immediately postoperatively, 24 h, 48 h, 3 weeks, 1 year, 2 years, 3 years, and 5 years postoperatively (see also participant timeline {13}). The main outcome time point will be the 1-year evaluation. There is no repeated measure design statistical comparison planned and the 1-year, 2-year, 3-year, and 5-year outcome will be evaluated separately to evaluate the effectiveness of the respective treatment constantly for patients.

The SF-36 (Short Form Health 36) consists of 36 questions and is a general health questionnaire that uses 8 different dimensions to make statements about the patient’s state of health. It includes statements about the following: vitality, physical functioning, bodily pain, general health perceptions, the physical role of functioning, social role of functioning, and mental health.

#### Participant timeline {13}

The participant timeline is shown in Fig. 2.

#### Sample size {14}

The Institute for Medical Biometry and Statistics at the University of Freiburg, Germany, provided biometric advice. The sample size estimate of this confirmatory study (superiority study) is based on the primary target value “Oswestry Disability Index (ODI).” The ODI is the gold standard of patient-related outcome measures (PROMs) in patients after spinal surgery [9, 12].

Null hypothesis: H0: *T*-*S* = *δ*

Alternative hypothesis: Ha: *T*-*S* > *δ*

Test statistic: *Z* = (*d*-*δ*)/*sd*

*T*: full endoscopic therapy; *S*: microsurgical therapy (standard/control); *δ*: “clinically accepted limit of superiority”; *d*: true difference in the mean between the two groups; *sd*: standard error of *d*

Calculation of the sample size:

$$N=2\times \frac{{\left({Z}_{1-\alpha }+{Z}_{1-\beta}\right)}^2}{\delta -{\delta}_0}\times {s}^2$$

*N* = number of cases per group; z1-α/1 − *β* = standard deviation for a one-sided 1-*α* or 1 − *β*; *δ* = true difference in the mean between the two groups; *δ*0 = clinically acceptable limit of the mean value difference (“relevance limit”); S2 = variance

With an expected difference *d* in the mean value between the two groups of *d* = 8 (maximum number of points 45 points in the ODI), an assumed standard deviation of *s* = 10, a clinically acceptable limit for the mean difference of *δ*0 = 4, a 5% level of significance (*α* = 0.05), an equivalent allocation to the groups (*k* = 1) and an assumed 10% drop-out rate, a number of cases of *n* = 87 per group, or a total of *n* = 174 is required to achieve a power of 80% (1 − *β* = 0.8).

#### Recruitment {15}

Recruitment will be performed at the Loretto Hospital Freiburg, Germany. Recruitment of the calculated sample size of patients will be feasible considering the high rate of spine surgeries at our hospital and our experience from the last 2 years. Our comprehensive cohort design further allows to include patients refusing to be randomized in a prospective parallel cohort, preventing selection bias. Additional strategies to increase recruitment include presenting the study on our hospital website, consultation of partner institutions, and providing information letters in the hospital's waiting rooms.

### Assignment of interventions: allocation

#### Sequence generation {16a}

Allocation (1:1) will be based on computer-generated randomization using a block size of 4 for sequence generation.

#### Concealment mechanism {16b}

Sequentially numbered, opaque, sealed envelopes (SNOSE) will be used to implement the allocation.

#### Implementation {16c}

An independent researcher will perform the generation of the allocation sequence and preparation of the sealed envelopes. Allocation (1:1) will be based on computer-generated randomization using a block size of 4 for sequence generation. FH will enroll participants and screen for eligibility. Hereafter, envelopes will be handed over to GL by the independent researcher, who will assign participants according to the allocation sequence.

### Assignment of interventions: blinding

#### Who will be blinded {17a}

The researchers analyzing the final data will be blinded.

#### Procedure for unblinding if needed {17b}

Not applicable as surgeons and patients will not be blinded.

### Data collection and management

#### Plans for assessment and collection of outcomes {18a}

BS and SU will be responsible for contacting the patients for follow-up visits to obtain the filled questionnaires. Laboratory markers and clinical characteristics (e.g., operation time, the hospital stay) are available from the hospital patient information system.

#### Plans to promote participant retention and complete follow-up {18b}

Patients will receive reminders on their preferred way (e.g., SMS, WhatsApp, telephone) in regular intervals discussed with each patient individually to improve the patient-doctor contact and retention to follow-up.

#### Data management {19}

The data acquisition and data management will be carried out as part of the study by BS and SU. The data evaluation and interpretation of the pseudonymized data will be carried out in the Loretto Hospital Freiburg, Germany. Data will be obtained from the in-house patient information system and pseudonymized at the Loretto Hospital, Freiburg, Germany, by applying a code to the data. The code is generated using the “encode” command in the STATA statistics software (Version 15, StataCorp. 2011, College Station, TX, USA) using a calculation process. The pseudonymized table contains an alphanumeric code generated from the patient identification number by a calculation process. Before this, the numeric patient identification number is converted from “numeric” to “string” format using the “tostring” command. The assignment table is stored in encrypted form in the Loretto Hospital in Freiburg and is only available to BS. The assignment table is encrypted using AES-256-bit encryption (Advanced Encryption Standard). With the pseudonymized table alone, no assignment to the patient’s personal data is possible without the aid of the assignment table.

#### Confidentiality {27}

At no time do third parties have access to the data. Every patient is informed about the storage and use of the data. The written consent of each patient included is obtained for the processing of the data for research purposes. The patient’s consent to the storage and processing of the data for research purposes is obtained for a specific study.

#### Plans for collection, laboratory evaluation, and storage of biological specimens for genetic or molecular analysis in this trial/future use {33}

Not applicable as only simple blood samples will be collected and directly analysis by the in-house laboratory.

### Statistical methods

#### Statistical methods for primary and secondary outcomes {20a}

Descriptive statistics will be applied to the data (mean, standard deviation, median, interquartile ranges, frequencies). The Shapiro-Wilk test will be used as a normality test. Normally distributed data will be analyzed using repeated measure ANOVA and paired *t*-tests, whereas non-normally distributed data will be analyzed using the Friedman and Wilcoxon tests. We will also generate and evaluate quotients of inflammatory parameters to evaluate their impact on PROMs. An initial screening of all outcome parameters will be performed with explorative statistical approaches and univariate analyses to evaluate which combination of parameters and quotients might be relevant for PROMs. A *p* < 0.05 will be considered significant.

#### Interim analyses {21b}

Interim results will be analyzed at 1 year, 2 years, and 5 years to allow constant evaluation of the surgical outcome for patients. Interim results will be communicated to all research study members. Cancelation of the trial or the decision to publish preliminary results of high relevance to the research community will be decided after an in-depth discussion and consensus with all research study members and the responsible institutional review board.

#### Methods for additional analyses (e.g., subgroup analyses) {20b}

Not applicable as we do not plan to perform subgroup analyses yet.

#### Methods in analysis to handle protocol non-adherence and any statistical methods to handle missing data {20c}

If lost to follow-up exceeds 20%, an intention to treat analysis will be performed, utilizing single imputation replacing the missing value with the mean of the respective variable.

#### Plans to give access to the full protocol, participant level-data and statistical code {31c}

The full protocol, pseudonymized participant-level data, and the statistical code will be provided to qualified researchers, health authorities, and funders on reasonable request.

### Oversight and monitoring

#### Composition of the coordinating center and trial steering committee {5d}

A regular internal meeting involving all research members (GL and FH, BS, SU) will take place (at least each month) to ensure quality monitoring. Non-regular meetings will be conducted where necessary.

#### Composition of the data monitoring committee, its role and reporting structure {21a}

As the therapeutical approaches are both established in our hospital and the outcome assessment regularly performed, and the data regularly included in our in-house patient information system, an external data monitoring committee is not involved. BS and SU will assess the outcomes and manage the data. A regular internal meeting involving the other research members (GL and FH) will take place (at least each month) to ensure quality monitoring. Research members (BS and SU) handling the data are independent of funders and sponsors and do not have a conflict of interest.

#### Adverse event reporting and harms {22}

Complications seen during surgery and the follow-ups will be assessed as secondary outcomes. These can include, for example, residual pain (VAS change ≤ 30% compared to the preoperative score), postoperative hematomas, fractures, and residual sensorimotor deficits.

#### Frequency and plans for auditing trial conduct {23}

Internal meetings will take place at least once a month involving all involved research members. External meetings will take place upon request of the institutional review board or the funders.

#### Plans for communicating important protocol amendments to relevant parties (e.g., trial participants, ethical committees) {25}

Protocol changes will be communicated to the institutional review board and the trial registry.

#### Dissemination plans {31a}

Results of this study will be published in scientific journals presented at scientific conferences.

## Discussion

We present a protocol for a comprehensive cohort trial involving both a randomized and a non-randomized study arm to compare the full-endoscopic approach for the treatment of lumbar disc herniation against the gold standard, the microsurgical decompression technique. As far we are aware, this is the most comprehensive study to be performed on this topic. One main advantage is that our protocol involves one surgeon who is specialized in both techniques. This approach reduces confounding bias associated with different learning curves or multiple surgeons. Furthermore, we decided to focus on patient-reported outcome measures as these are of high clinical relevance. The Oswestry Disability Index is widely used in patients with back pain. The inclusion of this outcome as our primary outcome allows future meta-analyses to reliably compare our study with other relevant studies on this topic. A further advantage of our approach is that we included a non-randomized study arm which allows us to evaluate the impact of randomization on study outcomes. Although laboratory markers are surrogate markers and might fail to show clinical relevancy as diagnostic markers for muscle damage, interleukin 6 is considered as an important mediator that can reliably evaluate the impact of injury [13]. Modern endoscopes allow high-resolution visualization of a broad surgical field. However, some cases are judged to be not adequately treatable with the full-endoscopic procedure during surgery and require a switch (“conversion”) to the microsurgical technique during surgery. This can represent a problem if occurred too often, as the allocation will change during surgery. We aim to count these cases as complications for the full-endoscopic technique and analyze them as full-endoscopic patients, although the microsurgical method was finally applied. Nevertheless, based on our surgeons” experience, this occurred rarely in the last year, and we assume that not more than 2–3 patients in our cohort will be affected.

## Trial status

The recruitment began on 14 January 2022. This is the first version of the protocol registered at The German Clinical Trials Register (DRKS), a German WHO primary registry, under the registration number: DRKS00025786. We plan to complete recruitment by 30 December 2025.
